# Promoting synergistic research and education in genomics and bioinformatics

**DOI:** 10.1186/1471-2164-9-S1-I1

**Published:** 2008-03-20

**Authors:** Jack Y Yang, Mary Qu Yang, Mengxia (Michelle) Zhu, Hamid R Arabnia, Youping Deng

**Affiliations:** 1 Harvard University, PO Box 400888, Cambridge, Massachusetts 02140-0888, USA; 2 National Human Genome Research Institute, National Institutes of Health, US Department of Health and Human Services, Bethesda, MD 20852, USA; 3 Southern Illinois University, Department of Computer Science, Carbondale, IL 62901, USA; 4 University of Georgia, Department of Computer Science, Athens, GA 30602, USA; 5 University of Southern Mississippi, Department of Biological Sciences, Hattiesberg, MS 39406, USA

## Abstract

Bioinformatics and Genomics are closely related disciplines that hold great promises for the advancement of research and development in complex biomedical systems, as well as public health, drug design, comparative genomics, personalized medicine and so on. Research and development in these two important areas are impacting the science and technology.

High throughput sequencing and molecular imaging technologies marked the beginning of a new era for modern translational medicine and personalized healthcare. The impact of having the human sequence and personalized digital images in hand has also created tremendous demands of developing powerful supercomputing, statistical learning and artificial intelligence approaches to handle the massive bioinformatics and personalized healthcare data, which will obviously have a profound effect on how biomedical research will be conducted toward the improvement of human health and prolonging of human life in the future. The International Society of Intelligent Biological Medicine (http://www.isibm.org) and its official journals, the International Journal of Functional Informatics and Personalized Medicine (http://www.inderscience.com/ijfipm) and the International Journal of Computational Biology and Drug Design (http://www.inderscience.com/ijcbdd) in collaboration with International Conference on Bioinformatics and Computational Biology (Biocomp), touch tomorrow's bioinformatics and personalized medicine throughout today's efforts in promoting the research, education and awareness of the upcoming integrated inter/multidisciplinary field. The 2007 international conference on Bioinformatics and Computational Biology (BIOCOMP07) was held in Las Vegas, the United States of American on June 25-28, 2007. The conference attracted over 400 papers, covering broad research areas in the genomics, biomedicine and bioinformatics. The Biocomp 2007 provides a common platform for the cross fertilization of ideas, and to help shape knowledge and scientific achievements by bridging these two very important disciplines into an interactive and attractive forum. Keeping this objective in mind, Biocomp 2007 aims to promote interdisciplinary and multidisciplinary education and research. 25 high quality peer-reviewed papers were selected from 400+ submissions for this supplementary issue of *BMC Genomics.* Those papers contributed to a wide-range of important research fields including gene expression data analysis and applications, high-throughput genome mapping, sequence analysis, gene regulation, protein structure prediction, disease prediction by machine learning techniques, systems biology, database and biological software development. We always encourage participants submitting proposals for genomics sessions, special interest research sessions, workshops and tutorials to Professor Hamid R. Arabnia (hra@cs.uga.edu) in order to ensure that Biocomp continuously plays the leadership role in promoting inter/multidisciplinary research and education in the fields. Biocomp received top conference ranking with a high score of 0.95/1.00. Biocomp is academically co-sponsored by the International Society of Intelligent Biological Medicine and the Research Laboratories and Centers of Harvard University – Massachusetts Institute of Technology, Indiana University - Purdue University, Georgia Tech – Emory University, UIUC, UCLA, Columbia University, University of Texas at Austin and University of Iowa etc. Biocomp - Worldcomp brings leading scientists together across the nation and all over the world and aims to promote synergistic components such as keynote lectures, special interest sessions, workshops and tutorials in response to the advances of cutting-edge research.

## Introduction

Establishing and maintaining productive collaboration between bioinformatics and genomics domains are critical for the shoulder-to-shoulder advancement of science and technology. The synergy of bioinformatics and genomics proved effective and powerful in promoting science, technology, medicine and the interdisciplinary research and education. Biocomp and the International Society of Intelligent Biological Medicine (ISIBM) aim to promote the synergistic genomics and bioinformatics research, therefore Biocomp 2007 received more than 400 high quality papers. Each paper was reviewed and ranked by at least 3 experts in the field. After a rigorous and unbiased review process, 27% of the papers were selected as regular research papers and 22% second-tire papers were accepted as “short” research papers that require/recommend authors to reduce their page-length to five pages. In addition, there are several invited keynote and tutorial lecture papers/abstracts from world top scientists in the fields. Program, Advisory and Steering Committee Chairs and Conference Co-Chairs have spent tremendous amount of time and efforts out of their tight schedule to this great academic event in evaluating 400+ papers and keeping the scientific standard high.

Worldcomp committees elected distinguished plenary keynote and tutorial lectures, given by world top scientists such as Dr. A. Keith Dunker of Indiana University - Purdue University, Dr. Jun S. Liu of Harvard University – Massachusetts Institute of Technology, Dr. Ruzena Bajcsy of University of California at Berkeley and Member of the National Academy of Engineering and Member of the Institute of Medicine of National Academy of Sciences, Dr. John Holland of University of Michigan, Dr. Mary Qu Yang of National Human Genome Research Institute – National Institutes of Health, Dr. Jack Y. Yang of Harvard University, Dr. Joydeep Ghosh of University of Texas at Austin, Dr. Howard J. Siegel of Colorado State University, Dr Patrick S Wang of Northeastern University and supported their cutting-edge research lectures to imbue all the 1,850 conference participants with new ideas. In addition, Biocomp 2007 hosted special genomics sessions focusing on specific genomics topics to promote the interdisciplinary and multidisciplinary education and research and fostered collaboration between the genomics and bioinformatics domains. Biocomp 2007 was evidently a large flagship international conference in the fields and received top ranking.

This *BMC Genomics* supplement consists of 25 peer-reviewed papers selected from the 2007 international conference on Bioinformatics and Computational Biology (BIOCOMP07), which is an international conference held simultaneously with a number of other joint conferences as part of the World Congress in Computer Science, Computer Engineering, and Applied Computing (WORLDCOMP, http://www.worldacademyofscience.org) in Las Vegas, U.S.A on June 25-28, 2007. WORLDCOMP is the largest annual gathering of researchers in computer science, computer engineering and applied computing. It attracted over 1,850 computer science and engineering researchers as well as computational biologists from 82 countries. The goal of the conference is to provide a forum in which researchers can present their research projects, exchange ideas in the research areas that interact and initiate the collaboration in their future research.

## Scientific themes, process of submissions and reviews

Bioinformatics and genomics play fundamental roles in our understanding and designs of biological systems and therapic medicine at all levels of organization, from molecular biology, life sciences to engineering and computer sciences. Bioinformatics and genomics are “bourgeoning out” fields that study the sequence with the development of algorithms, computational and statistical techniques, and theories to solve formal and practical biomedical problems.

Obviously, the Biocomp 2007 would not have achieved such a success without the hard work by contributors and organizers. Organizing such a major academic event in the fields is not possible without contributions from members of program, scientific review, advisory and steering committee. Thanks must be given to them for their professionalisms. We must express our sincere gratitude program and scientific review committee members for their high-quality timely evaluation of more than 400 full-length regular research papers. We must express our sincere gratitude to the program and scientific review committee members for their high-quality timely evaluation of more than 400 full-length regular research papers. We must extend our sincere thanks to all to the conference co-chairs, vice-chairs, session chairs, organizers and committee members for their dedication and professional services. In particular, Michelle M. Zhu, Youping Deng, Hamid R. Arabnia, Jack Y. Yang, Mary Qu Yang, Rattikorn Hewett, Yunlong Liu, Jianlin Cheng, Vladimir N Uversky, My Tra Thai, Yufang Jin and the scientific review committee members dedicated themselves for the scientific reviews.. Hamid R. Arabnia managed the paper submission system and handled various important organizing and academic affairs; Jack Y. Yang and Mary Qu Yang initiated the special genomics sessions with new components that are defined dynamically in response to specific needs of inter/multidisciplinary cutting-edge research and education, therefore, Mary Qu Yang, Jack Y. Yang and Hamid R. Arabnia initiated and arranged the organization of cutting-edge research workshops, keynote lectures, special sessions and poster presentations in addition to the traditional tutorial lectures. The Biocomp committee would like to acknowledge our appreciation of International Society of Intelligent Biological Medicine (ISIBM) for their academic support and co-sponsorship; we must express our sincere appreciation to the excellent professional services provided to the Biocomp by Isobel Peters at BioMed Central Ltd for the *BMC Genomics* supplementary issue.

Biocomp received submissions both from the presenters at the conference and from non-presenters. Submitted manuscripts were reviewed by at least three referees. The quality of each paper was evaluated based on the contribution to genomics and bioinformatics. The accepted papers in the specific issue covered a broad range of subject areas and can be mainly divided into the following categories:

### Microarray data analysis

Eight papers discuss novel mathematical or statistical approaches to analyze microarray datasets. Gu and Liu [1] proposed a Bayesian biclustering model, and implemented a Gibbs sampling procedure and illustrated that such Bayesian biclustering approach can effectively identify multiple clusters from gene expression data. Zhu and Wu [2] proposed a parallel computation-based random matrix theory approach to analyze the cross correlations of gene expression data in an entirely automatic and objective manner to eliminate the ambiguities and subjectivity inherent to human decisions. Yang *et al.*[3] conducted extensive physiological and transcriptomic studies to characterize Fur in Shewanella oneidensis, with regard to iron and acid tolerance response with their own microarray expression datasets. Xu *et al. *[4] developed novel graph-based methods to combine multiple microarray datasets to discover co-expression network modules related to cancer disease. Pirooznia *et al. *[5] compared various microarray classification methods including; SVM, RBF Neural Nets, MLP Neural Nets, Bayesian, Decision Tree and Random Forrest methods. Mao *et al.*[6] investigated the transcriptomic profiling of three yeast mutants lacking C2H2 zinc finger prote and found out that the gene expression patterns were dramatically different between wild type and the mutants. Gong *et al. *[7] studied the effect of explosive compounds such as TNT and RDX on the transcriptomic pattern of earthworms. Deng *et al.*[8] proposed an algorithms based on Integer Linear Programming to select a minimum number of non-unique probes for microarray experiment using d-disjunct matrices.

### Genome and sequence analysis

Li *et al. *[9] proposed an effective algorithm to enable rapid mapping of millions of oligonucleotide fragments to a genome of any length. They were able to achieve at least one order of magnitude speed increase over existing tools by using bit shifting operation.

Liu *et al.*[10] quantified the effects of recombination on populations by estimating the minimum number of recombination events in the history of a DNA sample. Two new algorithms were proposed for estimating the lower bound under the infinite site model. The new lower bounds can also be extended to allow for recurrent mutations. Yue *et al. *[11] extended current method GRAPPA for reconstructing phylogeny from genome rearrangements and develop a new method GRAPPA-IR to analyze gene rearrangement from chloroplast genomes with inverted repeat.

### Protein structure prediction and classification

Yang *et al. *[12] exploited machine learning techniques including variants of Self-Organizing Global Ranking, a decision tree, and a support vector machine algorithms to predict the tertiary structure of transmembrane proteins. Hecker *et al. *[13] developed a state of the art protein disorder predictor and tested it on a large protein disorder dataset created from Protein Data Bank. The relationship of sensitivity and specificity is also evaluated. Habib *et al. *[14] presented a new SVM based approach to predict the subcellular locations based on amino acid and amino acid pair composition. More protein features can be taken into consideration and consequently improves the accuracy significantly. Wang *et al.*[15] discussed an empirical approach to specify the localization of protein binding regions utilizing information including the distribution pattern of the detected RNA fragments and the sequence specificity of RNase digestion.

### Gene regulation elements analysis

Yang *et al.*[16] extended their previous work, which identified candidate bidirectional promoters in the human genome, to map the orthologous promoter regions in the mouse genome. It was shown that bidirectional promoters can be classified apart from other genomic features including non-bidirectional promoters. Chen *et al.*[17] developed an analytical method to identify a thermodynamic model that best describes the mode of transcription factor (TF)-TF interaction among a set of TFs for target genes. Wang *et al.*[18] conducted research to simultaneously identify transcription factor and microRNA (miRNA) binding sites from gene expression microarray database. Two models for predicting the most influential *cis*-acting elements under a given biological condition, and estimating the effects of those elements on gene expression levels are proposed.

### Disease classification using machine learning techniques

Yang *et al. *[19] developed a multi-task learning technique based on genetic algorithm to improve prediction accuracy of tumor classification by using information contained in such discarded redundant features. Experimental results demonstrated that this approach is effective and perform better than other heuristic methods. Liu *et al. *[20] developed a feature selection method to combines supervised learning and statistical measures for the chosen candidate features/SNPs to reconcile the redundancy information and, in doing so, improve the classification performance in association studies. A Support Vector based Recursive Feature Addition (SVRFA) scheme is also proposed to aid SNP-disease association analysis. Yang *et al.*[21] developed an intelligent decision system using machine learning techniques and markers to characterize tissue as cancerous, non-cancerous or borderline. These algorithms can detect microscopic pathological changes based on features derived from gene expression levels and metabolic profiles.

### Biological network construction

Hub proteins in a protein network can bind to many different protein partners to regulate and control a wide variety of physiological processes. Oldfield *et al. *[22] studied protein intrinsic disorder arising from structural plasticity or flexibility and illustrated how such intrinsic disorder can provide a means for hubs to associate with many partners. Jin *et al. *[23] presented their work on a nonlinear control and stability analysis of genetic regulatory networks. Such control scheme can make the genetic regulatory network to get to desired levels by adjusting transcriptional rates. This research the can also be used to design model-based experiments for gene expression profiles regulation.

### Genome and database search tools

Dai *et al.*[24] developed a visual editor for profile Hidden Markov Models (HMMEditor), which can visualize the profile HMM architecture, transition probabilities, and emission probabilities. As open-source software, it serves as a useful tool for biological sequence analysis and modeling. Vanteru *et al. *[25] introduced semantics enabled technique to link the PubMed to the Gene Ontology for ontology-based browsing. Latent Semantic Analysis (LSA) framework is used to semantically interface PubMed abstracts to the Gene Ontology for better search performance since semantics is introduced.

## Conclusion

As upcoming emerging fields, Bioinformatics and Genomics integrate science and engineering knowledge with modern state-of-art computing technology, which has so far slightly influenced the academic community and public consciousness. Bit new concepts and technologies are emerging at an incredibly pace and the synergy of Bioinformatics and Genomics proved powerful in the continuously evolving and emerging fields because the development of engineering and computer science approaches can be applied to both bioinformatics and genomics fields, the resonance and synergy of these two fields are enormous and will have significant impact on the advances of science and medicine. It is important to train the next generation of Bioinformatics and Genomics experts with up-to-date technologies and knowledge of advancement and development of these two emerging field. To these ends, Biocomp07 strives to create opportunities to promote science, technology and education for students, faculty, scientists and engineers in these new emerging fields and helps them better prepared for new initiatives and cutting-edge researches. Therefore, Biocomp07 offers a number of keynotes and tutorial lectures and Biocomp07 committee appreciates organizers performing their tasks in selecting best suitable papers in this BMC Genomics supplementary issue. Biocomp has attracted a wide scope of interests that significantly promote the science and technology with most updated cutting-edge technologies and breakthrough ideas win the open discussions, keynote and scientific exchanges among attendees to inspire innovations, novel ideas and scientific discoveries. Biocomp provided such unique platform and infrastructure to promote interdisciplinary and multidisciplinary research and education and this BMC Genomics supplementary issue is a product of part of Biocomp achievement.

## Future meeting

The international conference on Bioinformatics and Computational Biology is an annual conference. The next conference will be held in Las Vegas, USA on July 14-17, 2008. Information about the 2008 conference can be found on the web site http://www.worldacademyofscience.org/

## Competing interests

The authors declare that they have no competing interests.

## Authors' contributions

Jack Y. Yang, Mary Qu Yang and Michelle M. Zhu wrote the initial version of the paper, Hamid R. Arabnia and Youping Deng finalized the paper. All co-authors equally contributed to the paper, participated in the reviews of 400+ submissions and served as co-editors of the BMC Genomics supplement issue. Jack Y. Yang served as the International Program Committee Chair, Mary Qu Yang served as the Distinguished International Advisory Committee Chair, Hamid R. Arbania served as the Steering Committee Chair, handled the overall infrastructure of Worldcomp with 1,850 attendees and maintained the website of World-Academy-of-Science.org. Michelle M. Zhu and Youping Deng handled various editorial works and communications, and played important roles in the committee in evaluating and selecting papers from 400+ submissions. 
